# Compact Flexible Planar Antennas for Biomedical Applications: Insight into Materials and Systems Design

**DOI:** 10.3390/bioengineering10101137

**Published:** 2023-09-28

**Authors:** Dinesh Venkatachalam, Vijayalakshmi Jagadeesan, Kamal Batcha Mohamed Ismail, Manoharan Arun Kumar, Shanmugam Mahalingam, Junghwan Kim

**Affiliations:** 1 Department of Electronics and Communication Engineering, Kongu Engineering College, Perundurai, Erode 638060, Tamil Nadu, India; vijaya.jagadeesh09@gmail.com; 2 Department of Electrical, Electronics and Communication Engineering, School of Technology, Gandhi Institute of Technology and Management (GITAM), Bengaluru 561203, Karnataka, India; mohamedismailmk@gmail.com (K.B.M.I.); manokavi2011@gmail.com (M.A.K.); 3 Department of Electronics and Communication Engineering, Agni College of Technology, Chennai 600130, Tamil Nadu, India; 4 Department of Materials System Engineering, Pukyong National University, Busan 48513, Republic of Korea; shanmugam.mr3@gmail.com

**Keywords:** flexible antenna, wearable antenna, textile antenna, microwave imaging

## Abstract

Planar antennas have become an integral component in modern biomedical instruments owing to their compact structure, cost effectiveness, and light weight. These antennas are crucial in realizing medical systems such as body area networks, remote health monitoring, and microwave imaging systems. Antennas intended for the above applications should be conformal and fabricated using lightweight materials that are suitable for wear on the human body. Wearable antennas are intended to be placed on the human body to examine its health conditions. Hence, the performance of the antenna, such as its radiation characteristics across the operating frequency bands, should not be affected by human body proximity. This is achieved by selecting appropriate conformal materials whose characteristics remain stable under all environmental conditions. This paper aims to highlight the effects of human body proximity on wearable antenna performance. Additionally, this paper reviews the various types of flexible antennas proposed for biomedical applications. It describes the challenges in designing wearable antennas, the selection of a flexible material that is suitable for fabricating wearable antennas, and the relevant methods of fabrication. This paper also highlights the future directions in this rapidly growing field. Flexible antennas are the keystone for implementing next-generation wireless communication devices for health monitoring and health safety applications.

## 1. Introduction

Advancements in wearable electronics enables the realization of various body-worn applications. Wearable antennas that function as sensors have gained considerable research interest. These antennas can operate perfectly when worn on the human body. They are well suited for military applications, as they can provide soldiers with seamless and efficient communication capabilities while transmitting and receiving data. This can enhance the mobility and effectiveness of soldiers on the battlefield.

However, wearable antennas suffer from the problem of signal fading, whereby the signal strength at the receiving end decreases when the path length difference, due to the movement of the mobile terminal, becomes comparable to the signal wavelength [1,2]. Multipath fading can be reduced by employing antenna diversity. This can be achieved by placing two antennas half a wavelength apart; however, it is not feasible in most handheld devices and can only be adapted for large-scale body-worn antennas [3].

**Figure 1:**
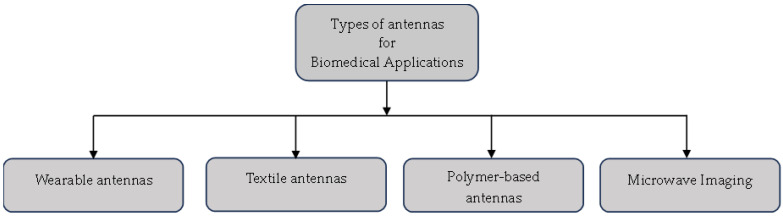
Types of flexible antennas for various biomedical applications.

Because of their functionalities and abilities, body-worn wearable technologies have sparked significant research interest in the past decade [4]. Figure 1 depicts the various types of flexible antennas for biomedical applications. They can be used in a variety of specialized fields that use body-centric communication systems, such as in the healthcare industry as a wearable tool to detect vital health problems in patients, recovery rooms, clinics, operation theatres, homes, and even on the move. Miniaturized antennas are also employed in military applications in camera and microphone modules for data transfer [5]. Furthermore, these antennas may be utilized for monitoring purposes by children, the elderly, and sportsmen such as healthcare systems, rescue units, and entertainment devices. Antennas are required to connect these technologies to other data collection stations for efficient data transmission. Similarly, some performance-based enhancements are required to create a clear function characteristic of a body-worn antenna [6]. Because these antennas operate in close proximity to the human body, and because of the loading impact of the lossy human body, the design of these antennas is more difficult. As a result, for applicability, these antennas must be user-friendly, robust, low in cost, light in weight, pleasant, maintenance-free, small, and require no installation, because the performance of the antenna varies while operating in free space and when put in other locations.

The water absorption and physical changes in the human body will have a substantial impact on the overall effectiveness of the antenna [7]. The design of these antennas varies depending on the operational frequency range, transmission strength, and the environment in which the antenna operates [8]. The overall performance of the antenna, however, is entirely reliant on the dielectric material that is utilized. In the antenna design, the dielectric material is selected based on its capability to sustain its characteristics in certain circumstances like bending, crumpling, and stretching repercussions. Conductive materials on the top and bottom layers of dielectric material have a high tolerance to deterioration due to mechanical deformation and resistance. The design of wearable antennas necessitates an understanding of the electromagnetic characteristics of the material that is utilized, such as permittivity, loss tangent, and substrate thickness [9]. Wearable materials are typically non-conductive and conductive textiles (electro-textiles). Non-conductive textile materials such as felt, silk, nylon, leather, wash cotton, denim, polymer, fleece, and paper are commonly utilized in the construction of wearable antennas to lower the weight and profile of the antenna [10,11,12,13,14].

A high-performance antenna system worn on/off the body and communicating nearby can be made possible by conductive fabric materials. Conductive textile (e-textile) materials are created by combining polymer threads or conductive metals with regular fabrics. Flectron, Zelt, pure copper, taffeta, and Sheildith are examples of conductive fabrics [15,16,17,18,19]. These materials are flexible and robust, making them suitable for body wear and making a substantial contribution to the wearable context. A lightweight and flexible substrate is required to develop a high-performance antenna system [20].

As previously stated, there are numerous ways to determine the dielectric characteristics of textile materials [21,22]. It must be guaranteed that the radiator does not have any negative effects on live organisms while developing these antennas. The substrate selection plays an essential role in the design of a wearable antenna in order to make the antenna robust for a specific application [23]. Textiles, in general, have a very low dielectric constant, which reduces surface wave losses and broadens the antenna’s impedance bandwidth.

The design factors considered for the implementation of wearable antennas are summarized below.

### 1.1. Selection of Materials

Wearable antennas must operate perfectly when they are worn on the human body. Wearable antenna technology mostly employs the wireless body area network (WBAN). Hence, the selection of a suitable substrate material (conductive and non-conductive) is a crucial aspect in the design of these antennas. The selected substrate must allow for the placement of the developed antenna in close proximity to a human body. Numerous researchers have conducted in-depth studies to select the ideal substrate material [24].

### 1.2. Human Body Interaction with the Antenna

Human tissues can react with incident electromagnetic waves because of the complex permittivity and conductivity of the tissues. The performance of an antenna is altered when it is placed near to a human body. The resonant frequency of the antenna is reduced owing to the higher value of the human body [25]. Moreover, the human body behaves like a lossy substance; hence, the tissues absorb the radiated energy and reduce the antenna’s gain [26].

### 1.3. Antenna Performance in Close Proximity to Human Body

When a wearable antenna is operated near the human body, the back radiation from the antenna is mostly absorbed by human tissues. Continuous exposure to the radiation might damage human tissues. All antenna sensors that are used in proximity to the human body must satisfy the specific absorption rate (SAR) standards defined by international organizations. The SAR value quantifies the amount of power absorbed per unit mass of human tissue. Therefore, while designing body-worn antennas, numerous factors such as the antenna’s radiation properties, the input impedance matching capabilities, and the power absorbed by the antenna should be considered [27,28].

### 1.4. Structural Variation of Antenna

An antenna that is worn on the human body undergoes bending, stretching, and twisting during the movement of the human body. Consequently, its radiation characteristics may degrade considerably, which affects the antenna’s radiation patterns, frequency tuning, and radiated energy [29,30].

### 1.5. Antenna Performance under Wet and Humid Conditions

Body-worn antennas that are fabricated from textile materials face certain operative challenges due to the presence of microscopic holes in the fabric. The microscopic holes can absorb moisture from the environment and increase the dielectric constant of the fibric material. This can significantly impact the antenna’s performance, thereby leading to effects such as frequency detuning, reduced radiation efficiency, and altered characteristics [31].

As the substrate plays a major role in determining the antenna’s characteristics, this study aims to highlight the recent substrates proposed for wearable antennas, with an emphasis on flexible antennas suggested for biomedical purposes. Additionally, it sheds light on the prospective avenues of its applications.

The primary difficulty with antennas worn on the body is ensuring that their characteristics remain constant under varying conditions like room humidity variations, body temperature and wet conditions [32].

Similarly, when many wearable antennas/sensors are employed in a single application, the location and distance between the sensors are the most important elements in improving the system’s performance.

A UWB health monitoring system tracks plenty of regular activities leading to a healthy lifestyle by employing several wearable sensors at varied locations and distances [33].

Furthermore, most wearable antennas are linearly polarized, and polarization mismatches arise owing to human body movement.

Wearable antennas work in close proximity to a lossy human body, which affects the antenna’s efficiency, driving point impedance, bandwidth, and gain. When electromagnetic waves are utilized for communication, part of the radiation is directed at the human body, penetrates through the skin, and is absorbed by the body, resulting in detrimental health consequences. Wearable antennas should comply with a high efficiency and a low specific absorption rate (SAR) in addition to bending and crumpling repercussions and flexibility. The amount of energy that is absorbed by the human body per unit mass is measured in W/kg as the SAR. This problem has been important for the past few years, and various regulating agencies have established a safe limit for the SAR for these antennas.

Various solutions have been offered in the past to overcome the aforementioned issues [34]. In [35], the authors created an ISM band wearable antenna by employing a pair of strip lines for feedline isolation and bandwidth augmentation. It was observed that the suggested approach’s major contribution was its ability to increase the bandwidth without significantly improving the SAR reduction.

To achieve a multiband response, the authors used the method of fractural slot loading. Furthermore, the increased bandwidth comes at the expense of structural complexity [36]. To lower the SAR of a meander line antenna for wearable applications, a truncated ground technique is adopted. The substrate, however, is a 1.6 mm thick FR-4, which is not suitable for wearable applications. Other SAR reduction strategies include changing the antenna feed point or direction, as well as inserting an EM wave absorber [37,38].

Additionally, PEC reflectors are employed as an additional antenna component to improve the effective radiation efficiency and lower the SAR of wearable antennas [39].

However, the low-profile property suffers when PEC reflectors are used. The SAR of antennas may also be reduced using ferrite sheets; however, this results in a greater integration cost and a bulkier volume, making the antenna unsuitable for body-worn applications [40].

The purpose of this review study is to summarize the current advancements in the field of wearable antennas for body-centric communication as well as other crucial elements. Section 2 describes the material selection for the development of wearable antennas. Section 3 illustrates the various types of antennas such as wearable, textile-based, polymer-based, and wideband antennas for microwave imaging applications. Section 4 concludes the entire article.

## 2. Material Selection

Conventional antenna materials might be unpleasant and are not suitable for body-worn antennas. To overcome this issue, an appropriate textile material may be employed in their design. Conductive (e-textile) materials have the properties of an imperfect and anisotropic electric conductor. As a result, these conductive materials have different conduction characteristics than standard conductors that are employed in conventional antenna designs [41]. The electrical and dielectric characteristics of some materials are also unavailable in the material library, which is a problem for antenna engineers [42]. Depending on the needs of the application, wearable antennas are placed on various regions of the body. As a result, the diverse motions of a human body cause the antenna design to encounter distinct structural deformations (bending). The performance of a wearable antenna is affected by bending deformation because bending can have a range of impacts on the structural characteristics of the antenna. Wearable antennas must behave consistently under different deformations in order to be considered functional. Therefore, it is crucial to research how bending affects the antenna’s overall performance.

Conductive (radiating element) and non-conductive (substrate) materials are the two most critical materials that influence the overall performance of wearable antennas. The dielectric characteristics, deformation (bending, crumpling, twisting, and stretching), sensitivity to miniaturization, and endurance in the external environment are used to select the substrate materials, while electrical conductivity is used to select the conductive materials. Prior to selecting a conductive material for the radiating portions, such as the feed line, patch, and ground plane, it is required to pick the most appropriate substrate material to support the layers. Wearable antennas will address the demand for comfort while also protecting consumers from dangerous radiation by adopting these materials. Furthermore, the material is chosen based on the aforementioned qualities to provide acceptable gain, high efficiency, and a suitable bandwidth [43,44]

### 2.1. Non-Conductive Materials

Non-conductive (substrate) materials or smart textiles can play essential roles in the construction of wearable antennas. To achieve higher efficiency, durability, and enough bandwidth, these materials must have minimum dielectric loss, thermal expansion coefficient, and relative permittivity [45]. The non-conductive materials used in the design have an effect on the overall performance of the wearable antennas. These materials not only sustain the radiating element, but they also have an impact on the antenna performance characteristics including return loss (S11), bandwidth, and efficiency [46,47].

Furthermore, the substrate material is crucial in terms of functioning, wear resistance, and manufacture. When choosing a substrate material, the permittivity, thickness, loss tangent, and flexibility should all be taken into account. The antenna’s performance, influencing the bandwidth, depends highly on parameters like material thickness and permittivity [48]. The loss tangent has an effect on efficiency. The lower the loss tangent value, the more efficient the antenna. Similarly, because of its flexibility, the antenna adjusts to the users. As a result, a suitable substrate material must match the design’s electrical and mechanical specifications. It is critical to analyze the characteristics of the substrate materials before constructing the antennas since the properties of the materials change based on the materials used and the frequencies employed [49].

### 2.2. Conductive Materials

Conducting materials are required for both the ground plane and the radiating element in all antennas. To maintain adequate antenna emission characteristics, wearable antennas must contain conductive radiating components and ground planes. Conductive materials (electro-textiles) are employed in the creation of wearable/textile antennas to serve as completely flexible and wearable antenna designs. The conductivity of the material determines the current that flows through the conductor, which is responsible for radiation. The conductivity of a substance is its capacity to allow for charges to flow freely through it. The material’s conductivity is measured in Siemens per meter (S/m). The perfect electric conductor (PEC) has unlimited electrical conductivity, making it excellent for antenna construction. In practice, however, all the materials have a limited conductivity. For example, the conductivity of an ordinary copper material is 5.8 × 10^7^ S/m. Conductive textile (e-textile) materials are employed for wearable antennas. As a result, materials with a higher electrical conductivity are preferred. As a result, the conductive materials that are utilized to construct wearable or flexible antennas must be very conductive. Therefore, the electrical characteristics of these materials must be characterized [46].

The electrical conductivity of the chosen material can have a significant impact on the performance of the antennas. Zelt, Flectron, Shieldit, and Taffeta are four different forms of electro-textile materials that have been documented in the literature and are commonly utilized as radiating elements and as ground planes. The conductivity of conductive materials may be estimated using Equation (1) below.

(1) $$\sigma =\frac{1}{\rho^{\tau }}$$

where ρ is the surface resistivity and *τ* is the thickness of the material. As a result, a low-loss dielectric material will be required as a substrate, and a highly conductive material will be required as a radiating portion for wearable antennas in order to provide effective electromagnetic radiation and acceptable performance.

## 3. Types of Antennas for Biomedical Applications

### 3.1. Wearable Antenna

**Figure 2:**
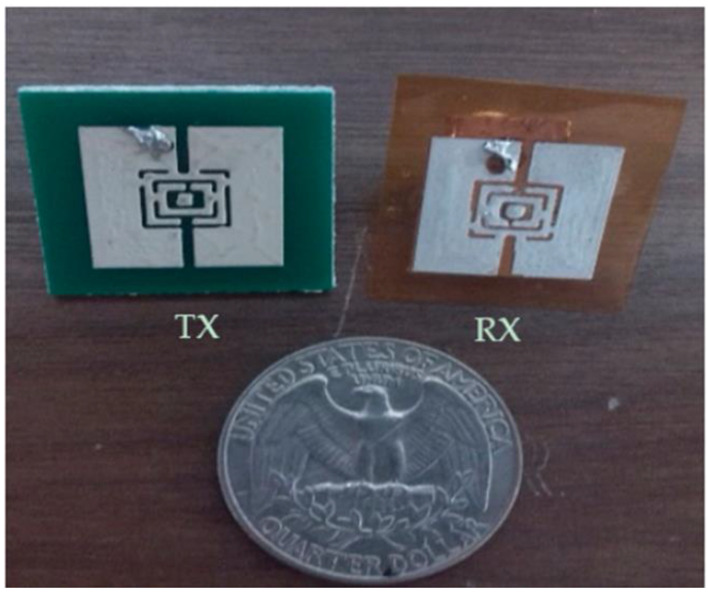
Capacitive electrode developed for the wireless monitoring of cardiac activity [50].

Advancements in wireless technology and on-body sensor design can revolutionize the traditional healthcare system by introducing wearable devices for healthcare monitoring [50]. Figure 2 depicts the wireless monitoring devices can continuously collect physiological data from an individual and provide valuable information regarding their overall health. The realization of such health monitoring systems can help to prevent diseases and effectively manage existing conditions, thus leading to reduced expenditure on human care. In this study, we review the recent developments in diagnostic non-invasive wireless technologies, particularly the devices and methods employed for examining blood glucose levels, blood pressure, respiratory movements, and cardiac activity. Additionally, we address the challenges regarding the on-body propagation of signals from multiple sensors.

The experimental analysis and design of body-worn antennas is integral to the implementation of body sensor networks (BSNs). Out of eighteen antenna designs, four designs were chosen, and their active and passive performances were assessed. The challenges encountered in this process, such as optimizing the antennas with impedance matching, transforming unbalanced signals to balanced signals, and characterizing antennas in different configurations (on-body and off-body), were investigated and resolved. This resulted in the development of a methodology for characterizing on-body antennas, considering the effects of the transceiver [51]. The proposed method adequately captures antenna performance in indoor environments, and the sole variation from the predicted performance stems from the influence of human body proximity. Generally, certain factors are considered when designing an on-body antenna, such as the application-specific customized design layout and the influence of the human body. Additionally, it requires a comprehensive simulation method that accounts for hardware mismatch effects.

In this study, we discuss the major challenges faced during the design and testing of on-body antennas in an anechoic chamber. This study builds on prior research by introducing three additional antennas and providing a more comprehensive explanation of the requirements analysis, design, development, and testing procedures. It introduces a systematic approach for actively evaluating the performance of a BSN antenna integrated with battery-powered devices, thereby yielding more realistic results. Additionally, this study examines the influence of the human body on the performance of an antenna through on-body experiments conducted in an anechoic chamber. Finally, it proposes and validates the use of a phantom to simulate and facilitate human experimentation with antennas.

**Figure 3:**
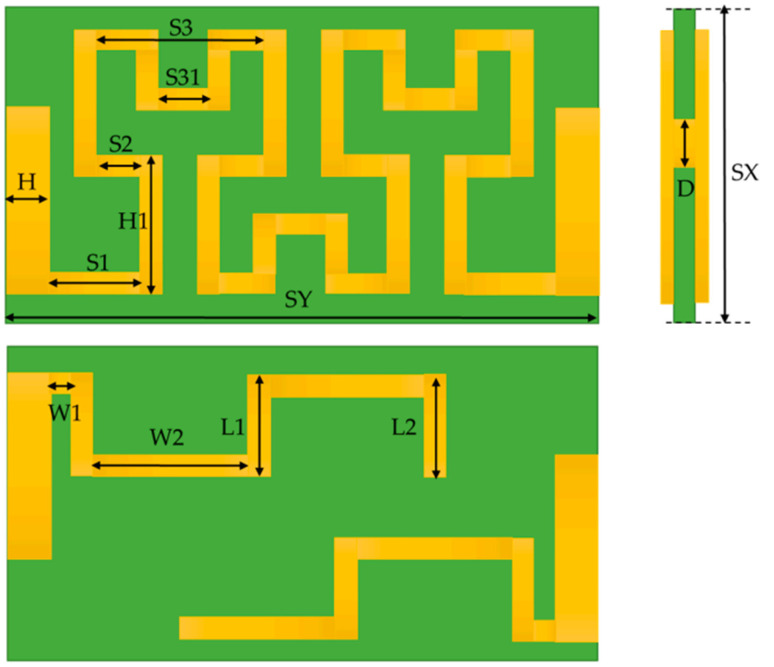
Dual-band fractal antenna for wireless data transmission [52].

A sequential array structure consisting of systematically arranged Minkowski fractal array elements was proposed in [52]. Figure 3 depicts the antenna leads were folded via a folding mechanism and positioned both at the top and bottom surfaces of the substrate, which effectively minimized the overall dimension of the antenna. The dimensions of the flexible antenna were 5 × 3 × 0.1 mm^3^, and a 0.1 mm thick polyimide substrate was used as the dielectric material. The fabricated antenna had three resonant frequencies, 2.4, 5.2, and 5.8 GHz, with a bandwidth of 100–1300 MHz. The antenna exhibited relatively low gains of 1.65 and 4.37 dBi at the resonant frequencies of 2.4 and 5 GHz, respectively, despite its small size. This antenna showcases practicality and holds a wide range of potential applications.

**Figure 4:**
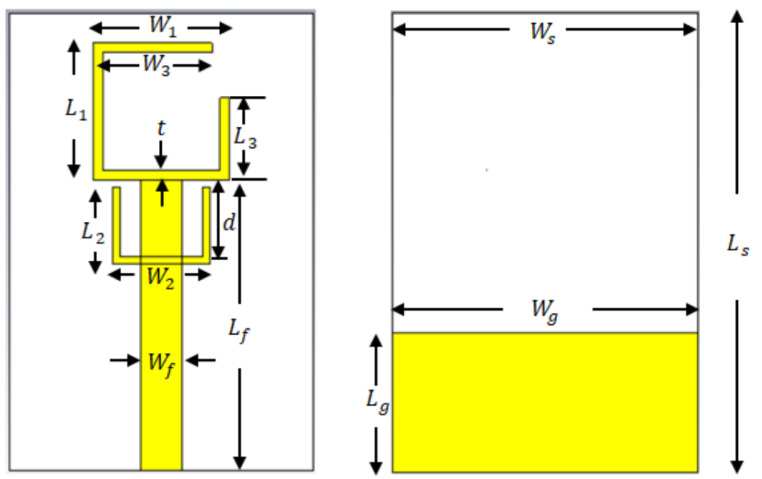
Triple-band antenna with split-ring resonator (SRR) and half-ring resonator [53].

A portable triple-band antenna was suggested for internet of things (IoT) devices in [53]. The radiating element had compact dimensions of 33 × 22 × 1.6 mm^3^ and exhibited multiple resonance is shown in Figure 4. The antenna was equipped with a square split-ring resonator (SRR) and half ring resonator, which enabled operations at 2.4, 3.7, and 5.8 GHz. The antenna was fabricated using a low-cost FR4 substrate and was suitable for wireless local area network (WLAN) and WiMax applications because of its compact structure. The design process involved changing the surface current distribution at each stage to obtain additional resonant frequencies. A conventional square patch was introduced at the initial stage. The ground was made partial, which reduced the surface waves and improved the radiation performance. To achieve dual-band resonance, a slot was introduced in the square patch, which converted it into a split ring. Finally, the addition of a second half-ring-shaped structure induced resonance at the third band.

Various periodic structures of radiating surfaces were proposed for realizing WBANs. Three different types of metamaterials were developed, namely electromagnetic band gap (EBG), artificial magnetic conductor (AMC), and metamaterial compounds. A compact composite right/left-handed coaxially fed dual-band antenna was fabricated and tested for GSM and wireless applications [54]. The AMC antenna developed in this study exhibits a directional radiation pattern and dual polarization and operates at 1.7, 2.5, and 3.8 GHz with gains of 2.1, 3.9, and 2.5 dBi, respectively. This study also developed an antenna with an EBC surface. This antenna is equipped with a spiral inductor and interdigital capacitor because of the shorting pins. The nearby patches reduce the antenna size and enables operations at three frequencies: 1.06, 1.8, and 2.5 GHz.

**Figure 5:**
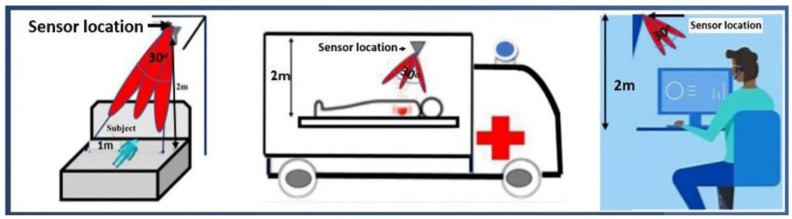
Schematic of a remote monitoring system [55].

The invention of multiple wireless communication techniques ushers in a new era of remote health monitoring systems that enhance the value of health services provided to patients is shown in Figure 5. Such systems help doctors and healthcare professionals to detect abnormal health conditions in patients at an early stage. BSNs are wireless sensor networks that are placed on patients to accumulate physiological information to monitor their health. They consist of small sensors that wirelessly transmit the collected health data to remote providers. Connected internet facilities are provided with mobility support to monitor patients continuously.

**Figure 6:**
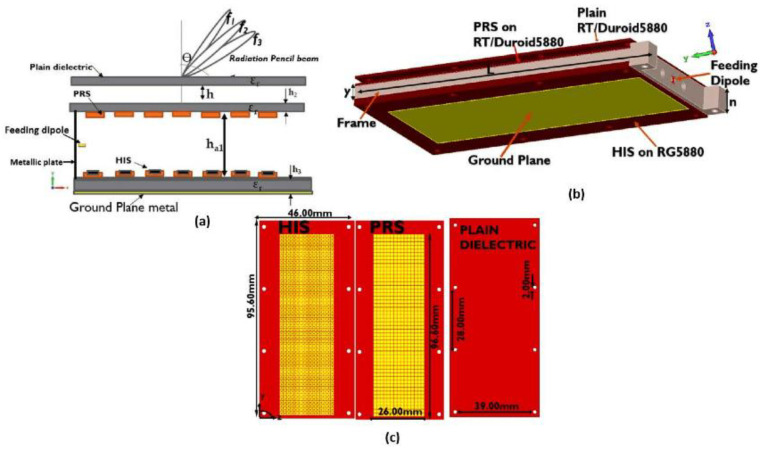
Structure of a leaky wave antenna (LWA): (**a**) geometry; (**b**) 3D view; (**c**) top and bottom views of the conducting surface [55].

Leaky wave antennas (LWAs) are a unique class of antennas that exhibit a highly directional radiation pattern is shown in Figure 6. Additionally, they can steer the emitted beam by varying its frequency [55]. When integrated with Doppler radar technology, these antennas emerge as the optimal choice for achieving remote vital sign monitoring. Their beam-steering capability enables them to monitor patient movements continuously without any mechanical rotations. LWAs possess a wide bandwidth and high directivity, which improves the signal-to-noise ratio and coverage in indoor environments.

**Figure 7:**
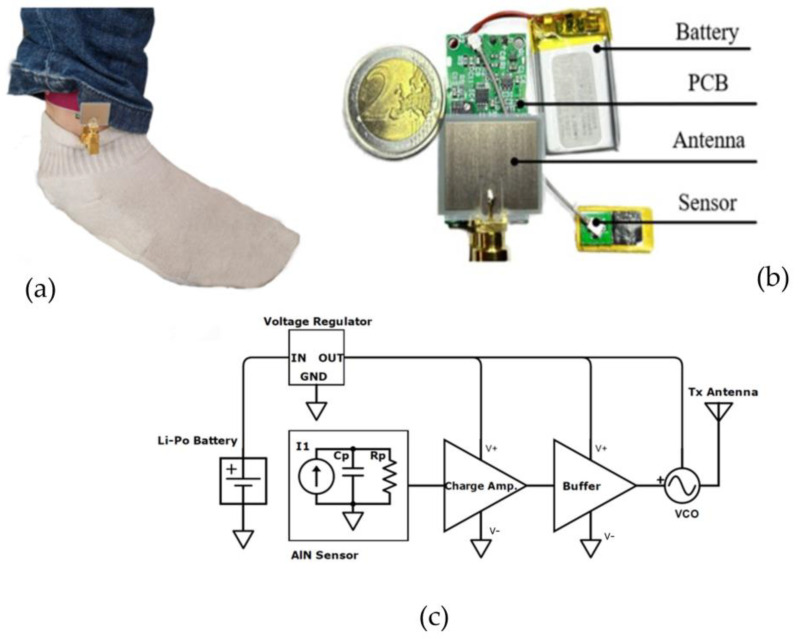
Overview of the proposed wearable device: (**a**) device placed on human leg; (**b**) list of components in the system; (**c**) circuit diagram of the system [56].

Figure 7 depicts an industrial scientific and medical (ISM) sub-6 GHz wearable sensor device for heart rate monitoring [56]. This frequency range provides the best trade-off in terms of the footprint, bandwidth, and communication range of all the bands that are currently available worldwide. Frequencies below 2.4 GHz are unviable as they increase the antenna footprints and decrease the range of reserved bands. Moreover, an issue of overpopulation is encountered owing to the multitude of communication protocols operating within this band. Frequencies above 6 GHz are ill suited for long-distance communications (mm-waves) owing to increased attenuations and coherence issues.

With the smartphone market reaching saturation, a new trend is emerging in the field of personal mobile devices, known as wearable mobile devices or simply wearables [19]. These devices come in various forms that are designed to be worn as accessories or clothing. Despite their small size, they are expected to continuously collect and transmit physiological data to enhance the quality of life. Consequently, wearable systems require improved communication security and reduce power consumption, thus prompting research efforts in these domains [57].

A previous study [58] presented and examined the design of a new compact antenna for multiband wireless applications. The antenna was fed by a uniplanar coplanar waveguide and had a small size of 25 × 25 mm^2^. It had a rectangular slot with three parallel stubs optimized for the radiator patch, as well as a T-shaped structure inverted in the ground plane. The final prototype of the antenna had three resonance frequency bands, 2.4–2.9, 3.7–5.2, and 5.7–6 GHz, with a return loss of less than −10 dB. The paper discussed the antenna configuration and design and its simulation and experimental results. It highlighted the compactness, simple feeding technique, and uniplanar design of the antenna, which simplify the integration of the antenna into various wireless devices.

A miniaturized button-shaped wearable antenna was designed for wireless signal transmission [59]. This antenna consisted of a transparent acrylic fiber substrate and a copper sheet as the radiating element, which was fed by a coaxial probe. The proposed button antenna operated between 5.25 and 5.58 GHz and exhibited a low gain. However, the antenna radiation characteristics can be improved using frequency selective surfaces on the radiating element, by which an average gain of 3.5 dBi can be realized. The placement of the superstrate also increases the bandwidth to 290 MHz.

The small size of millimeter-wave antennas makes them challenging to fabricate. Many applications, such as WiGig, employ the unlicensed V band (57–64 GHz). However, research on wearable millimeter-wave antennas is lacking. Karthikeya et al. proposed a wearable V-band antenna [60] designed as a button. This antenna consisted of a polyamide substrate with a thickness of 1.6 mm and diameter of 10 mm. The top of the button was a 0.01 mm thick metallic layer with a coaxial feed. The antenna used a partial ground and resonated at 59 GHz with a bandwidth of 1.1 GHz. A case study was conducted to examine an array setup of the antenna.

A wearable antenna with a small feeding network was discussed in [61]. The antenna used the aperture coupling technique. This design reduces the size of the rigid printed circuit board (PCB) that carries the electronic circuits and feeds the textile antenna, thereby improving user comfort. Additionally, it eliminates the need for probe feeding, which involves a single soldering point that is susceptible to breakage over time owing to user movements. A high manufacturing precision can be ensured using an aperture on the PCB. The antenna had a compact feeding network measuring 10 × 10 mm^2^ (0.0817 × 0.0817 λ02 at 2.45 GHz) and maintained a good performance in the ISM band.

**Figure 8:**
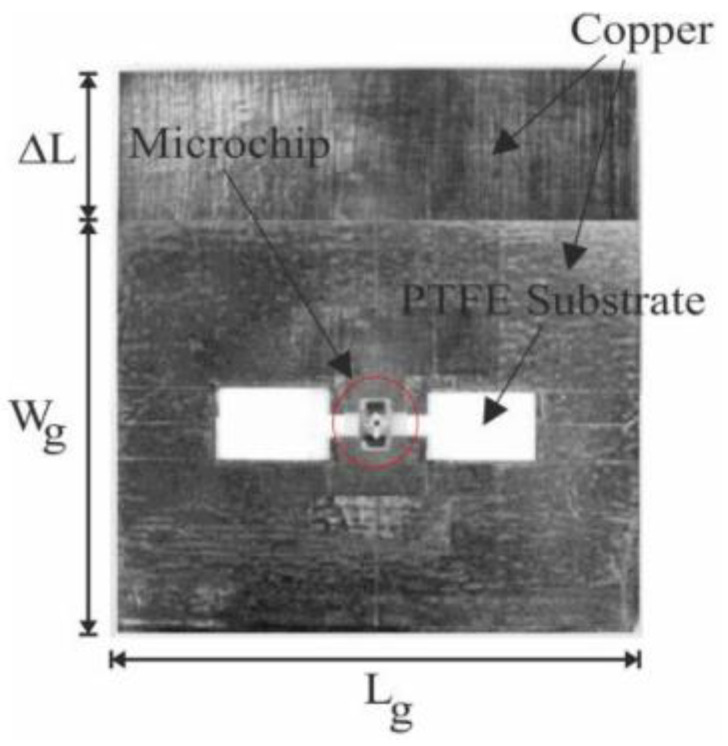
Geometry of the dumbbell-shaped ground plane [62].

In [62], a dumbbell-shaped ground plane for the antenna ground was employed to optimize the radiation patterns. In Figure 8 a comprehensive analysis of the antenna in terms of the electric and magnetic energies around the antenna borders was conducted [62]. Various ground-plane structures were proposed to analyze the energy density around the radiating surface and assess whether it withstands the coupling between the antenna and human.

Antennas employed in short-range near-field ultra-high frequency (UHF) radio frequency identification (RFID) systems are typically positioned close to the human body [63]. This implies that the designer must ensure that the antennas are robust and shielded from body coupling effects. For grounded antennas, a specific criterion can be used to determine the appropriate shape and size of the antenna ground plane. In [63], Near-field region characteristics of the grounded antenna on the human body are measured. From the observed SAR value, the robustness of the antenna against body coupling effects can be ensured without significantly altering the size of the antenna.

The design considerations for a UHF band card-type tag, which was enclosed in a packet and placed near the chest for applications on our university campus, were discussed in [64]. The FEKO simulation tool was used to design four different types of tag antennas that are highly insensitive to platforms [64]. Additionally, they measured the input impedances of the tag antennas using a test fixture and a two-port network analyzer to validate the simulation results and confirm the reliability of the simulation tool.

Recently, body-centric wireless communications have gained considerable interest. A small and efficient planar inverted-F antenna (PIFA) that operates at 2.45 GHz for on-body communications was introduced in [65]. The antenna consisted of two shorting structures and a folded ground plane, which enhanced impedance matching and reduced the antenna size. Hence, the antenna was compact and maintained a low-profile even when it was positioned in close proximity to the human body. The study examined the antenna’s performance near an arm phantom through simulations and experiments. The results from both methods align well.

A flexible antenna operating at two frequency bands was proposed for data transmission. The antenna had a low profile and a size of 35 × 20 mm^2^. It was fed using a co-planar waveguide (CPW) [66] and operated at 2.44 and 5.8 GHz in the ISM radio band. The antenna had a Rogers Ultralam 3850 substrate, which made the antenna highly flexible. The antenna’s compact size, flexibility, and dual-band resonance in the ISM band make it well suited for wearable applications. Table 1 depicts the various substrate materials used for wearable antenna design.

### 3.2. Textile Antenna

**Figure 9:**
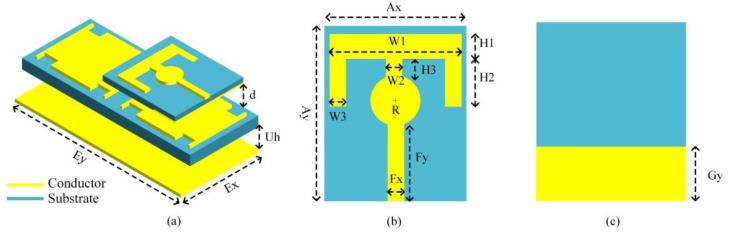
Wearable antenna: (**a**) 3D view with all layers; (**b**) front view of the antenna; (**c**) bottom ground plane [67].

A compact, flexible wearable antenna composed entirely of a textile material for the 2.45 GHz ISM band was discussed in [67]. It comprised a monopole radiator and a 2 × 1 EBG array, rendering it well suited for wristband applications [67]. The EBG unit cell was optimized for the desired operating band and further modified to maximize the bandwidth. The monopole radiator works in conjunction with the EBG layer to induce resonance in the ISM band with good radiation characteristics. Figure 9 shows the design of the antenna, whose dimensions are Gy = 10 mm, Ax = 35 mm, Ay = 52 mm, W1 = 28 mm, W2 = W3 = 4 mm, H1 = 7 mm, H2 = 13 mm, H3 = 6.42 mm, R = 5 mm, Fx = 2.5 mm, Fy = 26.12 mm, Ey = 35.4 mm, Uh = 5, and d = 1.9 mm, respectively. After fabrication, the performance of the antenna was tested in a free space and against human body loading. The antenna exhibited a bandwidth in the range of 2.39–2.54 GHz and had a compact size of 35.4 × 82.4 mm^2^. Experimental tests showed that the antenna maintained its performance when positioned near to the human body. The antenna exhibited a safe SAR of 0.297 W/kg at a 0.5 W input power, thus indicating that the antenna can be used safely in wearable devices.

**Figure 10:**
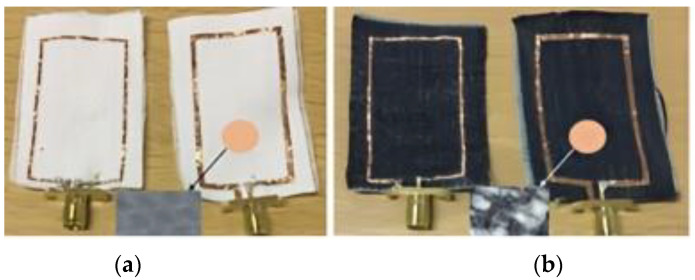
Textile antenna in which a slot is introduced for impedance matching on (**a**) cotton material and (**b**) jean material [68].

Another previous study [68] discussed a technique for enhancing impedance matching in a low-profile loop antenna using a high-impedance surface (HIS) structure, as shown in Figure 10. The loop antenna was designed to be wearable, and it is intended for healthcare applications. First, the study examined the impact of various textile parameters on the performance of the loop antenna, both through numerical simulations and experimental tests. Subsequently, it applied the proposed technique and validated the simulation results with the experimental results. Compared with a standalone loop antenna, the loop antenna with the HIS structure exhibited an improved matching performance in the 2.45 GHz band. In general, it also exhibited satisfactory far-field properties and a maximum gain of 6.19 dB. Additionally, the study investigated the effects of bending the loop antenna, both with and without the HIS structure, as well as its proximity to a modeled human arm. The wearable antenna with the HIS structure showcased commendable performance in these scenarios.

A small textile antenna was designed and tested for use in the ISM band at 2.4 GHz. The antenna utilized a rectangular slot/notch with a strip line inserted to form an inverted E-shape [69]. This design is simple, compact, and easily manufacturable using fabric materials. The antenna was 75% smaller than traditional antennas and maintained its performance even when it was bent. The equivalent circuit of each slot/notch and the strip line was determined and integrated to derive the overall equivalent circuit of the antenna. The results derived from the equivalent circuits were consistent with the simulation results. The antenna had dimensions of 30 × 20 × 0.7 mm^3^ and exhibited an impedance bandwidth of 15% and an efficiency of 79%. These findings indicate that the antenna holds significant potential for use in wearable systems.

A wearable microstrip antenna made from conductive textile fabric was introduced in [70]. It was designed for communications in multiple ISM bands. The antenna operated at three frequencies, 2.450 GHz, 4.725 Hz, and 5.800 GHz, and it was composed of a silver fabric patch and ground plane mounted on a flexible low-permittivity foam substrate [70]. A copper reference prototype was fabricated to validate the design. The experimental results from the two antennas show the expected resonances; however, some unexpected loss was observed, particularly at high frequencies. The simulation results for the antenna performance under different bending conditions demonstrate that the design is robust, with the resonant frequencies deviating within an acceptable range.

Recently, fabric and textile antenna designs have gained widespread attention owing to the growing demand for multi-frequency and multi-function antennas in smart clothing and future consumer-centric communication technologies [71]. The mode of fabrication and materials used play a crucial role in determining the performance of these antennas. A previous study [71] examined the influence of fabrication methods and materials on the performance of a rectangular microstrip patch antenna. The study aimed to realize antennas that are cost-effective, easy to integrate into systems, and capable of maintaining their performance when worn on the human body.

The performance of an antenna equipped with a textile substrate and an AMC was discussed in [72]. This study proposed a method of obtaining single- and dual-frequency resonances using two different textile materials, namely denim and flet. The antenna element was engraved on the textile materials using the electro-textile method to impart flexibility to the antenna. The hexagonal AMC structure acts as the reflector, which reduces the backward radiation and protects the human body during radiation. The antenna operated in the broadband frequency and was fed using a CPW.

The biological attributes of the human body, such as tissue properties and skin layers, play a crucial role in determining the propagation of radio signals through the human body [73]. A textile monopole antenna operating at 2.45 GHz was tested on different subjects to analyze its radiation effects. The results indicate that the antenna was resilient to changes induced by the human body: only 0.9% and 0.2% shifts in the impedance matching level and resonant frequency were observed, respectively, when the antenna was placed 10 mm away from the body. The antenna was composed of a ripstop and woven polyester fabric coated with copper and nickel. It had a thickness of 0.17 mm and weighed approximately 230 g/m^2^. According to the manufacturer, the surface resistance of the fabric was below 0.05 Ω/sq.

Antennas employed for healthcare applications usually operate in multiple band frequencies. A reconfigurable patch antenna is well-suited for biomedical applications. In [74], a compact button-shaped dual-band antenna for frequency reconfigurability was discussed. The proposed antenna operated in the 2.45 and 5.8 GHz unlicensed bands. Next-generation wearable textile antennas are expected to be body-worn radiating elements operating under the unlicensed frequencies of 2.45 and 5.8 GHz. The compact antenna was printed on a jean substrate with dimensions of 42 × 13 × 1.5 mm^3^, and it exhibited an omnidirectional radiation pattern. The study assessed the performance of the antenna in both a free space and human body surface. Owing to its compact size, this antenna may find utility in numerous applications within the smart wearable textile industry.

A planar inverted-F antenna (PIFA) is the most preferable radiating element for transmitting signals in portable wireless devices. In [75], the performance of a PIFA was evaluated for various distances between the human body and antenna and its scattering effects on human tissue. The study also investigated the near-field, electric, and magnetic-field distributions of the radiating patch. The antenna–body coupling was mitigated by adjusting the antenna ground plane, which enhanced the effectiveness of the antenna.

Several wearable antennas that are resistant to the body coupling effect have been reported in the literature [76]. To achieve this resistance, an antenna must maintain good input matching and efficiency even when positioned in close proximity to the human body. In [76], the efficiency of complimentary structures in coupling with the human body was evaluated and compared. Two body-worn antennas were highlighted: a printed patch antenna with a meandered ground and a meandered slot antenna with an ungrounded surface. According to our research, magnetic antenna designs, such as meandering slot printed antennas, are favored for wearable systems, even though similar structures are virtually comparable in non-wearable systems. Through careful design considerations, the susceptibility of these antennas to human body proximity can be mitigated substantially through careful design decisions.

**Figure 11:**
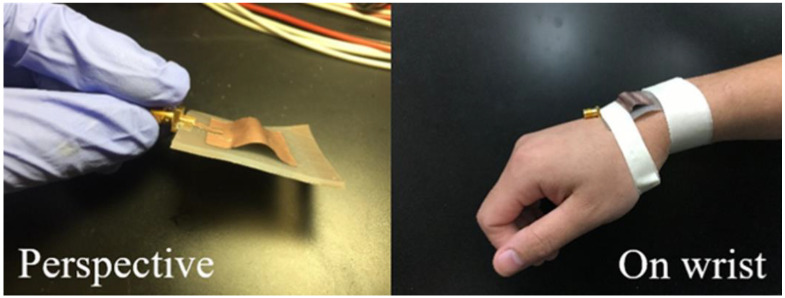
Wearable antenna worn on the wrist for bending analysis [77].

Wireless technology is integral to the advancement of flexible and stretchable electronics, which has gathered attention in view of addressing the increasing demand for devices that are compact, portable, and comfortable [77]. Microstrip antennas have emerged as promising alternatives in the realm of wireless technology, owing to their high flexibility and advanced bending electronics assembly. However, the flexible nature of microstrip antennas often entails a decrease in their conductivity and radiation efficiency. To address this issue, two designs were proposed: the stretchable mesh microstrip antenna and the arch-shaped microstrip antenna. Both antennas employ a silicone substrate, which is sandwiched between conductive materials. The resonance frequency of the antennas varies with respect to the bending condition. However, the variation depends on the strain of the antenna element. The resonance frequency of the meshed microstrip antenna decreases as the antenna strain increases. The strain of the arched antenna must be decreased considerably to increase its resonance frequency. Among the two antennas, the microstrip antenna exhibits a higher sensitivity to strain, with the resonance frequency exhibiting 3.35- and 1.49-fold increases. The proposed antenna is adaptable to ultra-wideband (UWB) bandwidth, and it rejects the high frequencies in the UWB range. Additionally, the antenna performs well when placed on the body, thus making it a promising candidate for wearable applications is shown in Figure 11.

### 3.3. Polymer Based Antenna

**Figure 12:**
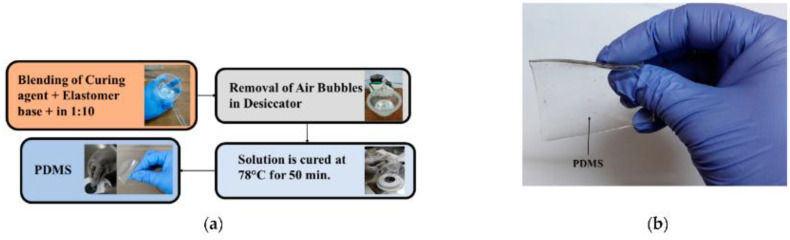
Polymer-based flexible antenna: (**a**) stepwise process of preparing polymer material; (**b**) fabricated PDMS material [78].

In [78], the properties of a silicone-based polymer polydimethylsiloxane (PDMS) substrate were examined to assess its applicability for realizing flexible and wearable antennae and sensors. First, the substrate was developed, ensuring that it satisfied the necessary requirements as shown in Figure 12. Subsequently, its anisotropy was examined through a bi-resonator experiment. The material showed a noticeable but moderate level of anisotropy: the dielectric constant and loss tangent exhibited anisotropy values of approximately 6.2% and 25%, respectively [78]. The anisotropic behavior was confirmed by the following: the dielectric constants in the parallel and perpendicular directions were approximately 2.717 and 2.570, respectively, with the former being higher than the latter by 5.7%. The dielectric properties of the PDMS substrate were found to depend on the temperature. Additionally, the study investigated the combined effect of bending the PDMS substrate and its anisotropy on the resonance properties of planar structures. These two factors induced opposing effects. Based on the experimental results, PDMS is a strong candidate as a substrate for flexible and wearable antennas and sensors.

Owing to rapid advancements in wearable electronics, flexible radio-frequency wireless antenna sensors have garnered considerable interest for their various IoT applications [79]. However, the miniaturization and flexibility of antenna sensors are impeded by device configuration and material issues. In [79], a wireless sensor that utilizes a microstrip antenna, which is composed of a copper ground plane, a PDMS dielectric substrate, and an MXene patch, is proposed. The optimized device is compact (40 × 40 mm^2^) and operates at a resonant frequency of 4.8 GHz, with a frequency shift range of 8% under different strains. Additionally, it is compatible with 5G communications. Additionally, the sensor exhibits excellent mechanical flexibility and maintains a stable response to repetitive stimulations. It exhibits a superior strain sensitivity of 20 compared with similar antenna sensors. Its resilience to human body proximity was tested by performing cantilever motion monitoring. The antenna exhibits satisfactory stability and sensitivity. It exhibits noticeably shifted resonant frequencies while maintaining a reflection coefficient of approximately −25 dB, indicating its potential for applications in the fields of healthcare monitoring, construction diagnosis, and IoT.

A smart pH measuring device can provide vital health information and has various applications in detecting infections, diagnosing diseases, and personalized medicine [80]. However, such devices are often expensive, have limited flexibility, and require large instruments to read the results, which render them ill suited for wearable, remote, and continuous health monitoring. In [80], a novel wearable sensor system, referred to as WB2F3D, is discussed. This sensor is a miniaturized and modular 3D-printed system. The system facilitates the on-demand, continuous, wireless, and real-time monitoring of pH levels. The sensors, electronic circuits, and antennas are printed using nanomaterials on flexible substrates, which facilitates multimaterial and multilayer printing in a cost-effective and efficient manner. The readout system is designed to be battery-free and flexible, enabling the wireless transmission of signals and data for continuous and real-time pH monitoring. The sensor system has a high sensitivity, specificity, repeatability, and reproducibility across various pH ranges. It is also mechanically flexible and biocompatible, as evidenced by a cell viability of at least 90%. The system successfully monitored the pH change in a wound model. The sensor system is expected to provide an integrated platform for accurate, on-demand, battery-free, wireless, and real-time monitoring of human health, bringing society closer to personalized medicine.

Interdisciplinary research focuses on routine healthcare objects to develop sensors that can monitor human biomedical parameters in an ergonomic and environmentally sustainable manner. In [81], a hybrid system that combines the solution-processed and conventional silicon technology is presented. A strain and pH sensor based on graphene ink is used, while a poly(3,4-ethylenedioxythiophene)–poly(styrenesulfonate) (PEDOT: PSS) sensor is used to detect humidity. These sensors are integrated into a demonstrator that is equipped with a silicon near-field communication transponder chip with logic processing and transceiver capabilities. The chip is bonded to a circuit board containing components that are inkjet-printed on a paper substrate, including the antenna, power supply, and sensors. This proof-of-concept study aims to meet the requirements of a future circular economy and provide a cheap, flexible, lightweight, and multi-functional electronic device.

In the past few decades, considerable efforts have been spent to develop simple and cost-effective methods for preparing superhydrophobic membranes using PDMS. In [82], a straightforward approach is presented to electrospin a PDMS membrane, using poly(methyl methacrylate) (PMMA) as a carrier polymer. The researchers investigated the effects of the PMMA concentration, PDMS/PMMA mass ratio, and key parameters of the electrospinning process (voltage and injection rate) to realize a superhydrophobic membrane that exhibits a high water contact angle (WCA). The highest WCA (163°) was achieved for the membrane surface that was electrospun with a solution containing PDMS, PMMA, tetrahydrofuran (THF), and N-dimethylformamide (DMF) in a mass ratio of 1:1:8.88:9.48. The process was conducted at an applied voltage and injection rate of 11 kV and 0.1 mm/min, respectively. The superhydrophobic PDMS/PMMA membrane was then used to conduct a membrane distillation process for desalination, and a high permeation flux of 39.61 L/m^2^ and an excellent salt rejection rate of 99.96% over a 24 h period were achieved.

Another previous study [83] discussed the development and analysis of a wideband antenna based on a PDMS composite. The metallic parts of the antenna were formed by incorporating conductive fibers into the PDMS composite. The performance of the antenna was compared with that of a traditional antenna composed of conventional dielectric materials. Both antennas exhibited a good matched bandwidth; however, the PDMS composite antenna showed reduced radiations towards the broadside direction, particularly at high frequencies. The antenna exhibited a matched bandwidth of 59.9%, covering a frequency range of 3.43–11.1 GHz. Additionally, the antenna’s flexibility was tested under different bending conditions. The wideband behavior was well maintained with a maximum variation of 1%.

A polymer-based flexible antenna that operates at three different frequency bands (5, 5.8, and 6.6 GHz) for WLAN and WBAN applications was developed in [84]. The antenna is fed by a CPW and has dimensions of 50 × 40 mm^2^. The antenna uses a PDMS substrate with a dielectric constant of 2.65 and a loss tangent of 0.02. The antenna has a rectangular slot on the ground and a single circular SRR on the patch. The SRR structure assists in achieving the desired frequency notching characteristics, compact size, minimal losses, and reduced backward radiation when the antenna is used near the human body. The proposed flexible antenna showcases a stable performance and a low SAR. The antenna performance was also evaluated under various moisture and bending conditions. The simulation results are consistent with the measured data.

Modern wearable health monitoring systems utilize multiple biosensors embedded in wireless devices [85]. This necessitates new antenna design requirements to ensure the reliable transmission of vital signs. Hussain et al. introduced a flexible, compact, and ultra-low-profile dual-band antenna. Its design is suitable for wearable and flexible telemedicine systems, as well as WBANs. Using the inkjet technology, the antenna is printed on a 50.8 μm polyimide Kapton substrate and is fed by a CPW. The proposed design offers advantages such as compactness, a light weight, wide bandwidth, high efficiency, and high mechanical stability. Additionally, the antenna’s performance was evaluated under bending and rolling conditions to simulate real-world usage on curved surfaces. The results indicate that the antenna is highly resistant to performance degradation resulting from bending. The proposed design is a viable option for the intended application owing to its favorable radiation characteristics, simplified fabrication process, cost-effectiveness, and excellent physical properties.

Another study [86] focused on the potential of integrating a highly conductive fabric into composite materials via an efficient fabrication technology. It aimed to fabricate a flexible composite antenna with high radiation performance. The process involved embedding a thin sheet of a conductive fabric into a composite laminate made of E-glass fiber mat and epoxy resin. The antenna elements were precisely machined via laser etching. The antenna exhibited a radiation efficiency of over 70% across its operating frequency range, with satisfactory radiation patterns and gain. The composite antenna performed well under different bending conditions. This study demonstrated the promising potential of integrating efficient antennas into various devices and objects made of composite laminate materials. The proposed fabrication technology was validated through the design and fabrication of a UWB antenna, and its performance was compared with that of a reference antenna comprising a copper sheet printed on the same composite dielectric substrate.

Another study [87] proposed a novel approach of integrating an ionic polymer metal composite (IPMC) actuator with an RFID tag antenna to achieve frequency reconfiguration in the UHF band. The IPMC actuator is shaped like a movable flap and is used to effectively tune the resonant frequency of the tag. To improve the deflection of the IPMC actuator, a two-layer crenelated structure and a surface patterned electrode are employed to restrain back relaxation. The IPMC actuator can move in two directions, which facilitates two-degree frequency tuning. The numerical and experimental data show that the displacement of the IPMC actuator tip can be increased by up to 266% using the two-layer crenelated structure. Using the IPMC actuator, the resonant frequency of the tag antenna can be adjusted by as much as 35 MHz, effectively correcting any frequency deviation caused by the unintended placement of the antenna on an object. The performance of the tag antenna was evaluated using a portable commercial RFID reader. The antenna exhibited good frequency reconfiguration, a broad tuning range, and long read distances.

PDMS is a versatile elastomer that possesses exceptional optical, electrical, and mechanical characteristics, rendering it highly suitable for various engineering applications [88]. Its biocompatibility has made it a popular choice in the biomedical field. Consequently, the soft lithography technique, which primarily utilizes PDMS, has been widely adopted for the rapid prototyping of micro and nanostructures. This technique has significantly contributed to advancements in microfluidics, electronics, and biomedicine. Another study [88] provided an overview of PDMS properties and commonly used PDMS treatments, emphasizing their applicability to the aforementioned fields. Additionally, it examined specific PDMS applications such as biomedical microchips, the replication of cardiovascular flow, and medical implants.

A new UWB antenna was developed for wearable applications in the 3.7–10.3 GHz band [89]. This antenna is designed to be flexible and resistant to human body loading and physical deformation. It has a size of 80 × 67 mm^2^ and is composed of a simple microstrip structure with two modified arc-shaped patches as the main radiator. The antenna also includes a full ground plane on the opposite side of the substrate to prevent interference from biological tissues and back radiation towards the human body. To ensure flexibility and durability, the antenna is fabricated using conductive fabric embedded into a PDMS polymer. The simulation and experimental results reveal that the antenna exhibits promising performances in both free-space and in vitro on-body cases. This is the first UWB antenna with a full ground plane that can withstand the harsh conditions that are typically encountered with wearable applications.

A flexible UWB antenna using a PDMS substrate was proposed in [90]. The conducting layers are composed of a 0.193 mm thick copper foil, and the overall thickness of the antenna is 1.886 mm. The antenna is designed to cover a frequency from 1.5 GHz to above 15 GHz. The performances of the antenna at specific frequencies (1.8, 2.4, 3.6, 4.2, 4.8, 5.2, and 5.8 GHz) were analyzed and compared. The SAR at these frequencies was evaluated and was found to be below the recommended value of 1.6 W/Kg. The performance of the antenna was assessed in terms of various parameters such as the reflection coefficient, VSWR, impedance, gain, surface current distribution, far-field radiation pattern, and the SAR. This study also discussed the use of a 1.5 mm thick PDMS layer as the substrate and a 0.193 mm thick copper sheet as the conductive layer for the radiator and ground. The performance of the antenna with and without bending was evaluated. The SAR and far-field analysis were conducted using layered phantom and human phantom models to determine the suitability of the antenna for body-worn applications.

The abrasive jet machining (AJM) of PDMS is either slow or impossible at room temperature because PDMS can absorb the energy of the impacting particles. In [91], cryogenic AJM was employed to increase the material removal rate; however, its efficiency depended on the mechanical performance of the PDMS samples. The samples were tested for compressive strength, hardness, X-ray diffraction, and linear expansion coefficient according to GB standards. The compressive modulus, ultimate compressive strength, and hardness of the samples were in the ranges of 17.31–1160.1 MPa, 160.25–224.50 MPa, and 43–90.67 ShD, respectively. They exhibited a coefficient of thermal expansion (CTE) of 247 × 10^−6^ 1/K at different temperatures from 103 K to room temperature (RT = 298 K) to 123 K. The results show that as the temperature decreased, the failure mode of PDMS changed from ductile to brittle, with evident brittle characteristics at 123 K.

**Figure 13:**
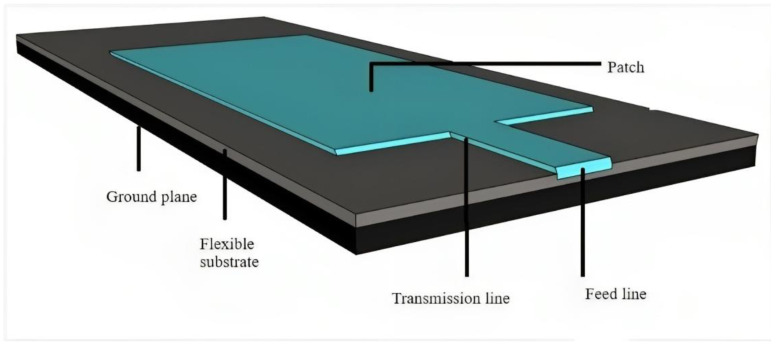
Flexible RFID tag system [92].

Another study [92] summarized the bending capabilities of flexible polymer substrates for general IoT applications. It explored the use of various flexible materials such as polymers, plastics, paper, textiles, and fabrics in wearable sensors is shown in Figure 13. It provided a chronological order of the flexible materials used in the past few decades and discussed their flexibility, bending ability, and resistance to deformation. In the future, IoT is expected to rely on wireless connectivity and support a wide range of technologies. Therefore, flexible materials with bending capabilities are crucial for realizing wearable IoT devices. The study examined commonly used polymer substrates and compared their physical, electrical, and mechanical properties. Additionally, it investigated the impact of bending on the radiation performance of antenna designs based on polymer substrates. It focused on the use of flexible materials such as polyimides (PI), polyethylene terephthalate (PET), PDMS, Polytetrafluoroethylene (PTFE), Rogers RT/duroid, and liquid crystal polymer for realizing flexible antennas in IoT applications. The study investigated the effects of bending and folding on radiation characteristics such as S-parameters, resonant frequency deviation, and impedance mismatch with the feedline of the microstrip antennas. These flexible polymer substrates hold promising potential for future wearable devices and general IoT applications.

**Figure 14:**
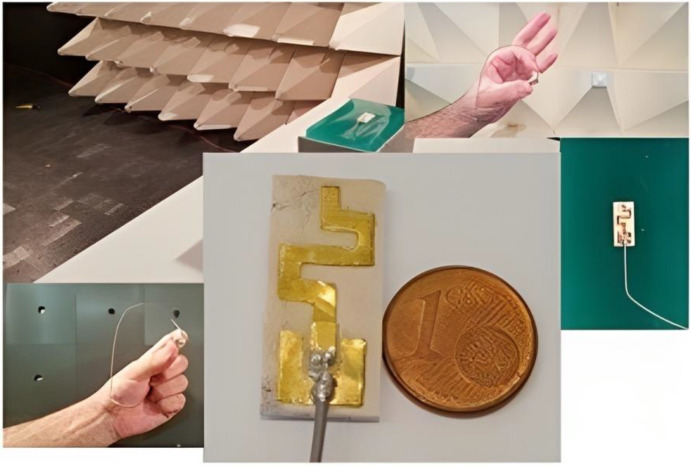
Fabricated flexible antenna on a semisolid muscle phantom [93].

Figure 14 is the compact flexible antenna was specifically designed for body-worn devices. To ensure the comfort of use and antenna flexibility when the antenna is in contact with the human body, a substrate made of natural rubber filled with TiO_2_ was developed [93]. The antenna was miniaturized using the quadratic Koch curve. The design, optimization, and characterization of the antenna were conducted using a human body model. The performance of the antenna was evaluated in terms of in-body and off-body wireless communication capabilities. The results indicate that the maximum telemetry range of the antenna for in-body and off-body communications exceeded 80 mm and 2 m, respectively. Additionally, the highest SAR value shown was 0.62 W/kg. With its compact dimensions (12 × 26 × 2.5 mm^3^) and low manufacturing cost, this antenna is well suited for health telemetry applications. Compared with other flexible antennas, the proposed antenna demonstrates smaller dimensions, better radiation efficiency, and higher gain when placed on a human body model.

**Figure 15:**
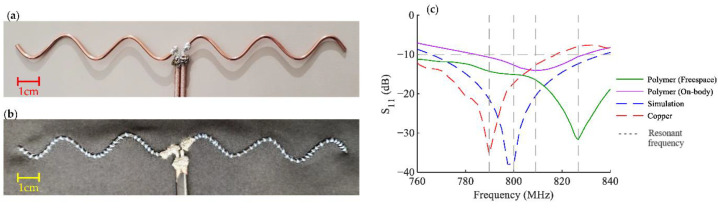
Fabrication and comparison: (**a**) copper metal antenna; (**b**) polymer antenna; (**c**) comparison between the return losses [94].

A modified sinusoidal half-wave dipole antenna sensor that can be used for strain sensing applications was discussed in [94]. Figure 15 is an antenna with improved version of a sensor that was previously introduced for respiration monitoring. The study examined the electrical and radiative characteristics of the sinusoidal antenna and the effects of various geometrical factors. It provided a design approach and equations to estimate the geometrical parameters according to the desired electrical specifications. The results show that the sensitivity of the antenna sensor can be up to 5.5 times higher than that of the previous generation. The use of a conductive polymer material for the fabrication of the antenna can render the antenna more flexible and durable compared to a glass-based antenna.

### 3.4. Antennas for Microwave Imaging

Microwave imaging is an efficient method used to diagnose cancer in its early stage. However, the efficiency of a microwave imaging system depends on the effectiveness of the antenna used for transmitting and receiving the microwave signals. Moreover, the antenna should be flexible. Extensive research has been conducted on designing a flexible antenna and increasing its efficiency to save the lives of people. An early detection of cancer can significantly enhance the chances for recovery, potentially saving numerous lives. In India, almost 2.5 million cancer patients have been registered, with 1 lakh new cases added annually. In 2018, 7 lakh deaths occurred owing to cancer. According to the Indian Council of Medical Research, India is likely to record over 17 lakh new cancer cases with over 8 lakh deaths by 2022. Breast cancer is the most prevalent type of cancer in women. These statistics underline the necessity of developing new methodologies for the early detection of breast cancer. Currently, image processing techniques, such as mammography, magnetic resonance imaging, and ultrasound, are available for breast-cancer diagnosis. To improve the efficiency of early breast cancer detection, a portable breast cancer detection device using the microwave imaging technique can be designed and developed. It involves designing a flexible wearable metamaterial antenna operating in a UWB frequency range and machine learning techniques. In addition, a flexible wearable metamaterial antenna can be used as a wearable device to address the limitations of the current detection technologies, which involve radiations that are painful and detrimental to the human body [63,95,96,97].

**Figure 16:**
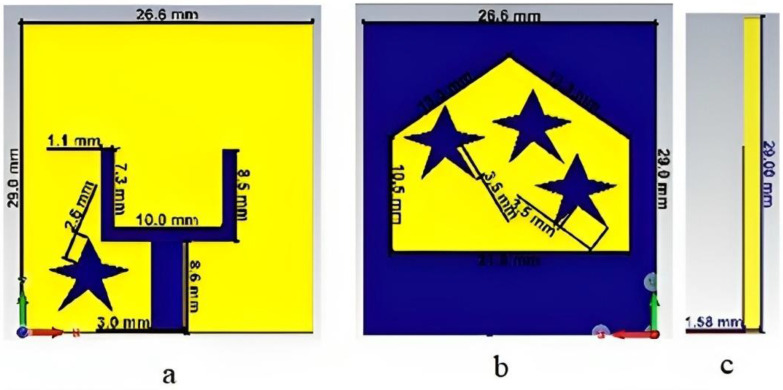
Ground plane and star patch: (**a**) U-shaped ground plane; (**b**) star patch on the top plane (**c**) side view of the antenna [98].

A U-shaped slot for ultra-wideband microwave imaging applications was proposed in [98]. The antenna consists of a tapered slot and star patches located on both the top and ground planes. The star patch located at the side of the feed line improves the impedance matching for the operating frequency band. The addition of a rectangular slot at the U-shaped slot improves the resonance at the lowest frequency, as shown in Figure 16. The tapered section containing the star shape patches at the bottom layer increases the antenna bandwidth from 3.8 to 10.1 GHz. A heterogenous phantom mimicking a human breast, with tumors introduced within the breast tissue, was developed and experimentally tested. The radiation characteristics of the antenna were evaluated in terms of the fidelity factor. Fidelity factors of 91.6% and 91.2% were observed for the face-to-face and side-by-side orientations, respectively. This implies that the antenna has good directional radiation.

**Figure 17:**
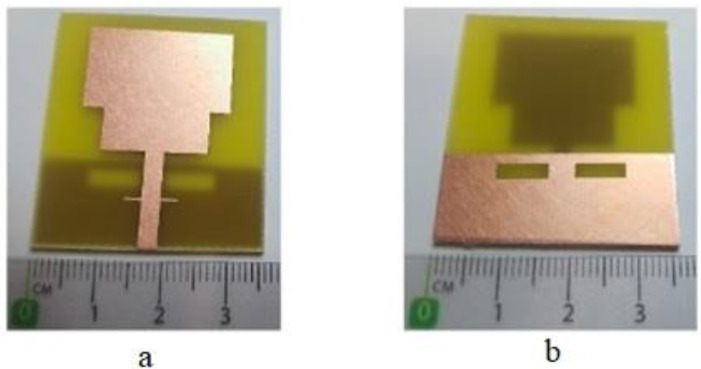
Fabricated planar antenna: (**a**) top view; (**b**) back view [99].

**Figure 18:**
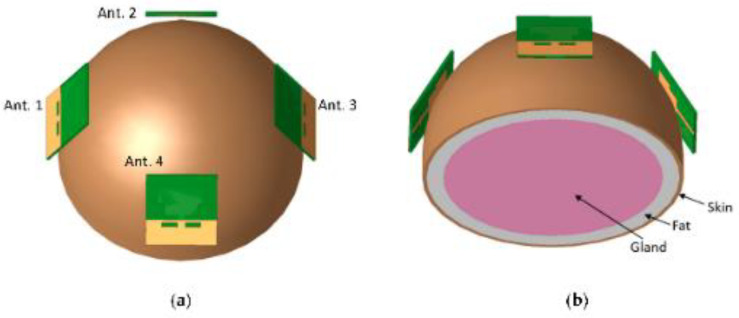
Simulated four-antenna elements around the human phantom: (**a**) top view of antenna arrangement and (**b**) layers of human model [99].

A printed monopole antenna was designed with staircase steps at the edge of the feed line, as shown in Figure 17. This antenna has dimensions of 40 × 36 × 1.6 mm^3^ and uses an FR4 substrate [99]. The operating frequency range and resonance can be varied by altering the monopole edges. The dimensions of the antenna were derived using the general transmission line model. Increasing the length of the current flow path in the antenna layout more easily simplifies the operation of the antenna in the low frequency despite its miniaturization. In the proposed antenna, one step is cut at the edge near the feed line and a slot is made at the bottom plane, which introduces impedance matching in the operating frequency range. The monopole antenna operates between 2.7 and 11.4 GHz and has a moderate gain in the range of 1–6.5 dBi. Figure 18 depicts the four-monopole antenna surrounding the human phantom model to study the performance of the antenna on a human body.

**Figure 19:**
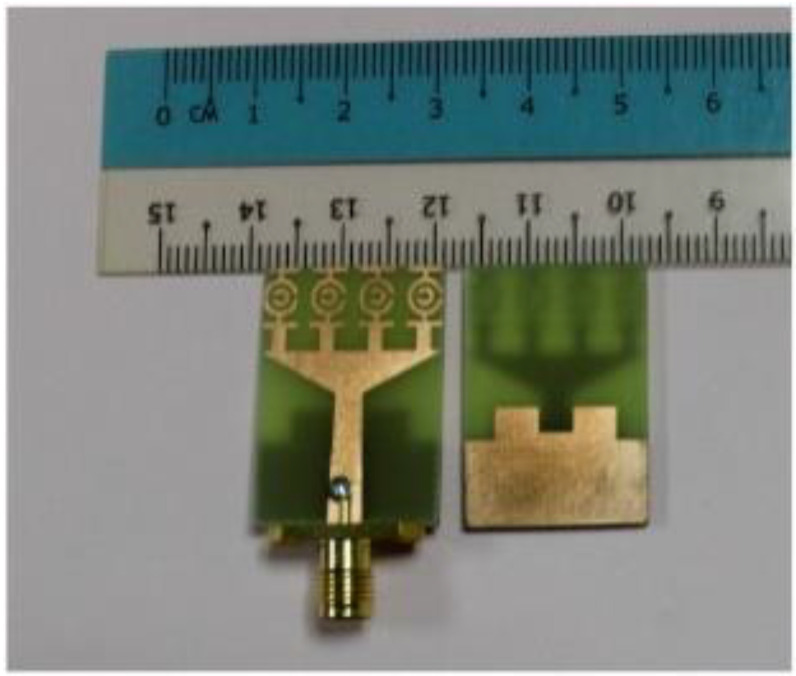
Fabricated UWB antenna with 4 metamaterial unit cells [100].

Metamaterials are remarkable engineering novelties, and they are shown in Figure 19. They can exhibit rare electromagnetic properties such as a negative refractive index. Negative-index or left-handed metamaterials and double-negative metamaterials are types of metamaterials that simultaneously exhibit negative permeability and negative permittivity. Their invention has paved the way for the development of microwave devices such as antenna sensors. Further, they can be used as regeneration systems in microwave imaging. The unit cell is a complementary SRR structure printed on a 1.6 mm thick FR4 structure [100]. The proposed SRR unit cell exhibits peak transmissions at 8.9 GHz, which indicates left-handed characteristics. One-element and four-element metamaterials have been proposed for UWB applications. A defective ground structure introduced in the ground plane optimizes the surface current in the patch antenna, which increases the operation frequency from 3.1 to 10.6 GHz.

**Figure 20:**
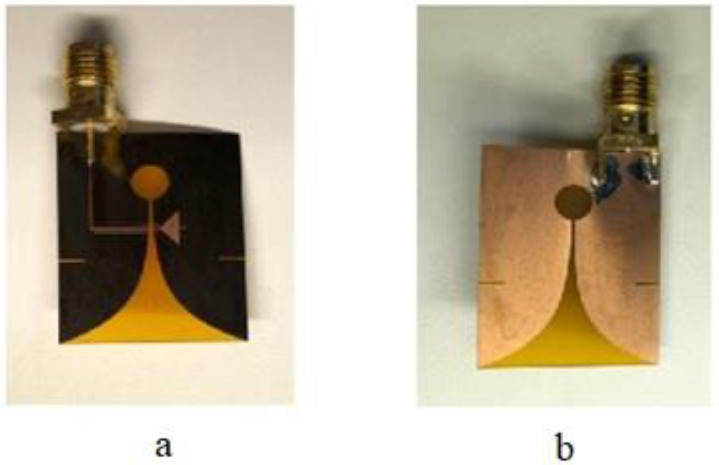
UWB Vivaldi antenna for microwave imaging applications: (**a**) bottom layer with feed line and (**b**) top layer radiating taper section [101].

In [101], a modified, small, flexible Vivaldi antenna with dimensions of 25 × 20 × 0.1 mm^3^ was proposed and examined, and it is shown in Figure 20. Two antenna designs were examined; one has a resonant frequency at 4.4 GHz, and the other has a resonant frequency at 9.4 GHz. The antenna was fabricated via the conventional PCB manufacturing method and uses a flexible material (polyimide) with a dielectric constant of 3.5 and a thickness of 0.1 mm [101]. With an array of nine identical 4.4 GHz antennas, a microwave radar-based imaging toolbox was employed to detect and pinpoint tumor cells inside a layered phantom model. The simulation results show that tumors of various sizes and positions within the phantom model can be successfully identified and located using the proposed method. The proposed microwave imaging technique is a viable method for the early diagnosis of breast cancer, and the proposed Vivaldi antenna is the most suited for breast imaging applications.

**Figure 21:**
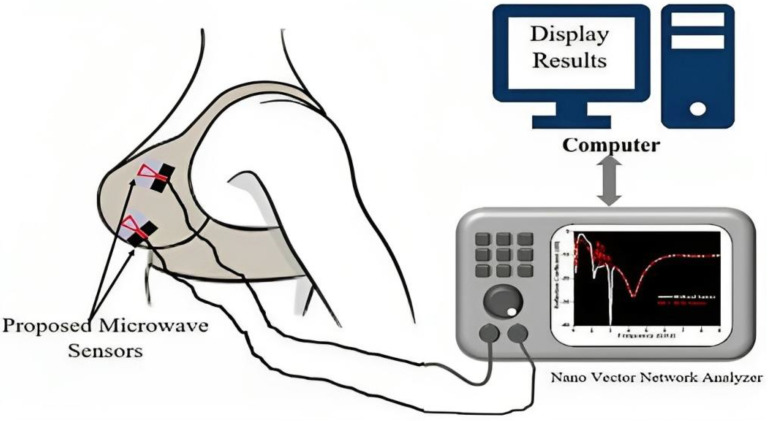
Wearable antenna with a microwave imaging system [102].

In [102], a biodegradable textile material and a conformal microwave sensor equipped with a circularly polarized antenna are presented, which has diverse medicinal applications. The proposed sensor consists of a monopole antenna printed on a textile substrate. The antenna has an overall size of 33.5 × 33.5 mm^2^ (0.2 λo × 0.2 λo) and is fed using a CPW as shown in Figure 21 [102]. A silver conductive fabric forms the radiating area and bottom ground, which are then stitched on a 2 mm thick cotton substrate. A slot is introduced into the radiating element to increase the bandwidth from 1.8 to 8 GHz. Near-field microwave scanning was employed to investigate both the on-body and off-body (air) performances of the proposed Circularly Polarized Microwave Sensor (CPMS) near human models with and without malignancies. The axial ratio is one of the major characteristics of a circularly polarized antenna and provides an additional degree of accuracy for data processing and cancer diagnosis. To ensure safe operations, the radiation characteristics of the device were recorded, along with its SAR. Consequently, the number of data samples increased, which helped to improve the detection efficiency. The proposed device was designed to assist women with the early detection and continuous monitoring of breast cancer in the convenience of their homes.

**Figure 22:**
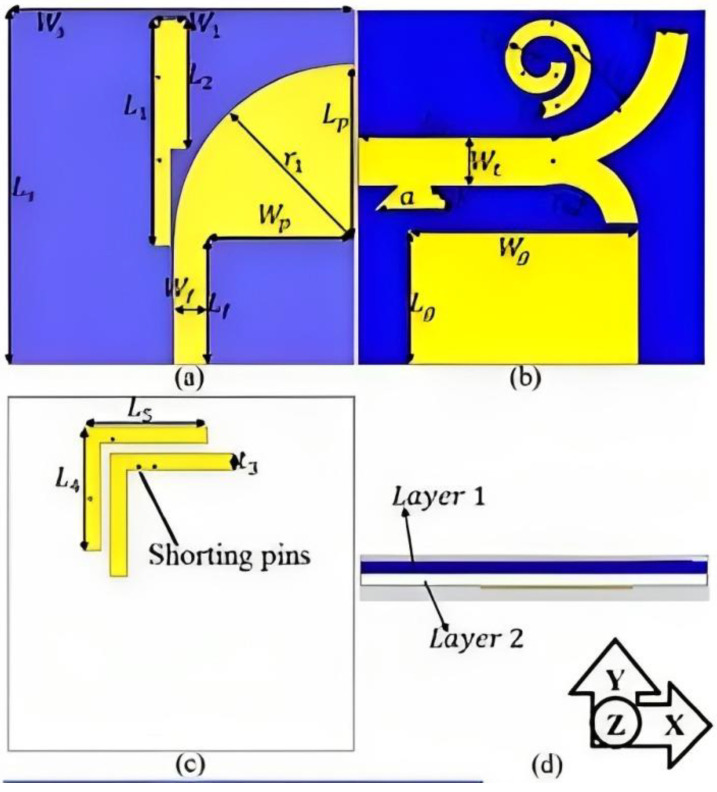
MIMO antenna: (**a**) front view; (**b**) first layer back view; (**c**) second layer front view and (**d**) side view of the antenna [103].

In [103], a small, wearable dual-polarized MIMO antenna is described. Dual polarization is achieved using tree-shaped and leaf-shaped antennas, which are horizontally and vertically polarized, respectively. The coupling effects at the desirable resonances are eliminated by implementing stubs, a parasitic spiral, and shorting pins [103]. The ShieldIt conductive textile is used in conjunction with a dual-layer substrate. The substrate has a felt layer and denim layer, whose dielectric constants are 1.8 and 1.2, respectively, with thicknesses of 0.9 and 0.5 mm, respectively. To obtain three additional cells, the dual-port MIMO unit cells were rotated 90° and separated by 2 mm, as shown in Figure 22. The antenna bandwidth satisfying the SAR standards for 1 and 10 g of tissues is between 4.8 and 30 GHz. After combining the shorting pin and spiral parasitic loadings, an isolation exceeding 18 dB was attained and a reduction in mutual coupling was observed. Both the on-body (breast) and free-space performances of the antenna were evaluated via near-field microwave imaging. Based on the appropriate operation frequency, low SAR, high fidelity, and high accuracy in tumor location, the proposed dual-polarized MIMO structure is a respectable option for breast tumor imaging.

Several numerical techniques were reported for antenna miniaturization based on patch antenna loading with slots, lumped elements, and shorting posts. The slots introduced are of various shapes, and the lumped elements, based on similar reactive components, are designed at the resonating frequency. The etching of various slots facilitates the reduction in the antenna size for modern wireless communication [104].

Antenna miniaturization is accomplished by fabricating a patch structure on a dielectric substrate with a dielectric value that is higher than that of the patch. However, this drastically increases the current distribution and leads to surface wave effects, which degrade the patch antenna characteristics such as the impedance bandwidth, radiation characteristics, and radiation efficiency [105].

Currently, UWB antennas are the most preferred antennas for non-destructive testing and microwave imaging. UWB antennas operate in the frequency range of 3.1–10.6 GHz, which encompasses low-frequency to very-high-frequency signals; hence, the use of UWB antennas produces images with better resolution. A device for obtaining the three-dimensional scan of a breast model was developed in [68]. The device employs a confocal microwave imaging algorithm to illuminate the breast organ and record details regarding tumors, skin, and healthy tissue [106].

Employing notch cutting in the ground plane reduces the effects at low frequencies. Impedance matching is provided for the input feed from the finite ground plane, and slots are introduced in the radiator. This prompts the antenna to radiate in the UWB frequency range [107]. The cancerous and healthy tissues are differentiated using a hybrid imaging modality in the microwave range and its performance is assessed using the Debye testbed setup [108]. A UWB monopole antenna with multifractal edges provides an improved impedance bandwidth across the operating frequencies [109]. Transverse electromagnetic horn antennas were tested using a compressed heterogeneous breast model for near-field microwave imaging [110]. Breast cancer detection based on UWB microwave imaging is used for early detection; however, the method is hindered by drawbacks due to complex algorithms. Machine learning methods assist in detecting cancer cells of smaller sizes, which may improve the accuracy of the results [111].

Recently, digital mammography has been adopted for breast cancer detection. Digital mammography is almost similar to the traditional method of film screen mammography. However, the latter involves compressing and positioning the breast organ over the mammography machine in a manner that may be painful for the patients. Digital mammography primarily aims to detect breast abnormalities. Compared to the conventional method of mammography, digital mammography provides breast images with a better resolution at lower radiation levels. However, it suffers from high false positive and negative rates. This necessitates additional examinations to confirm cancer and higher expenses for the early diagnosis of breast cancer. A flexible wearable patch antenna with a jean substrate is proposed in [112]. This antenna operates at 5.8 GHz.

The conductivity of the electro-textile material and its surface impedance was measured. Furthermore, the SAR analysis was conducted. The antenna exhibited increased bandwidth and efficiency. A metamaterial antenna was fabricated using a low-cost FR4 material, and the antenna characteristics were evaluated by applying metascreens over the patch.

In [113], the use of the above antenna for wireless applications is discussed. The body-centric performance of various conventional wearable designs, textile antenna designs, conductive materials, and fabrication methods were analyzed to assess their potential for wireless applications. The effect of bending and on-body performance were also assessed.

In [114], a radar-based UWB microwave imaging technique based on the dielectric properties of the human breast tissues is investigated. In [115], the performance of miniaturized flexible antennas placed at various positions on the breast is analyzed. A moderate dielectric constant can be achieved by varying the concentration of raw materials. The results were obtained from a healthy subject who used a wearable bra equipped with the antenna, and the results were analyzed to assess whether the performance of the antenna array was sufficient for breast tumor detection. In [116], a novel microwave sensor is proposed for breast cancer detection. The sensor exhibits improved performance in diagnosing cancer cells and higher image resolution compared to conventional antennas.

Conventional antennas are designed and fabricated on PCBs using substrates such as FR4, Rogers, and PTFE. If these antennas are used in wearable applications, they may obstruct and prevent reactions with the human body during cancer diagnosis [117]. Such antennas and the associated RF circuits are sealed as they are not suitable for all environmental conditions and may undergo corrosion from perspiration. They also suffer from mechanical stress. There are several types of wearable antennas, including microfluidic antennas with injection alloys, polymer-embedded antennas, embroidered antennas, 3D-printed antennas, and inkjet-printed antennas [118]. Fabric embroidered antennas can be fabricated by stitching suitable conductive threads directly on a clothing material. Further, another study [119] discusses the use of a polymer-embedded conductive fabric to achieve a low permittivity for on-body networks. UWB antennas operate in the frequency range of 3.1–10.6 GHz, which encompasses low-frequency to very-high-frequency signals; hence, it provides images with higher resolution [119,120,121,122,123,124,125]. The performance of various biomedical antennas is compared in Table 2.

Certainly, the scientific community, the technical community, and the clinical community need to work closely together to figure out solutions that synergize with one another in order to successfully develop and implement wearable antennas in the medical field. Each of these groups plays an important role in ensuring that technical breakthroughs are successfully incorporated into applications that are used in the actual world of healthcare. The authors in [126] illustrate microwave radiation and therapy in their depiction of the current state of affairs and of the medical devices for the next generation.

## 4. Conclusions

The research on wearable antennas focuses on developing towards low-cost, easy-to-fabricate BAN systems for medical applications and remote monitoring. This review begins by providing an overview of the different substrate materials and techniques used for wearable antenna fabrication. Substrate materials such as polymer, graphene, PDMS, textile materials, and microfluidic materials are discussed. Substrate materials play major roles in determining the coupling between the antenna and the human body and the characteristics of the wearable antenna under wet and humid conditions. Hence, the substrate material must be chosen carefully to ensure the robustness of the antenna’s characteristics against all environmental conditions.

The industry and the scientific community are becoming more interested in flexible wearable antenna sensors. Due to this, the following research problems and critical topics are provided for future studies in this field:

- Increasing the accuracy and effectiveness of the present measurement and manufacturing processes;
- Releasing new yarns and conductive textiles on the market that have increased conductivity or decreased resistance;
- Introducing new flexible materials for clothing that can be embroidered or new suggested production methods;
- Introducing novel body-operated antenna sensors based on substrate materials used for various applications.

For the impactful real-world application of wearable antennas, a further area of research is still required to be carried out in the field of materials science and system engineering. To enhance the performance of antennas as sensors in biomedical applications, new materials including conductive ink on textile substrates and graphene substances can be used. These novel materials are interesting components for the next generation of antenna design.

Flexible wearable antennas have gathered considerable attention from both the industry and scientific communities. This may lead to some research challenges. Researchers may focus their efforts towards increasing the accuracy and effectiveness of the current measurement and manufacturing processes. Developing innovative approaches can enhance the conductivity of yarn and textile materials.

Novel flexible materials can be used to fabricate wearable antennas for better performance. Future studies can focus on enhancing the performance and reliability of antennas used for medical and industrial applications. Further investigations can be conducted on textile-, microfluidic-, polymer-, and graphene-conductive-ink-based antennas to increase the performance of wearable antennas integrated to BAN systems. Advancements in wearable technology can facilitate the realization of diverse applications in the medical field.

## Figures and Tables

**Table 1:** Types of substrate materials commonly used for wearable antennas.

Type of Substrate	Dielectric Loss	Dielectric Constant	Thickness (mm)
Cotton/polyester	0.02	1.6	2.8
Woolen felt	0.02	1.6	3.5
Fleece fabric	—	1.25	2.56
PEN	0.025	2.9	0.125
Cordura	0.0098	1.1–1.7	0.5
Felt	0.02	1.3	1.1
PDMS-MCT	0.015	3.8	—
PET	0.008	3	0.14
Liquid crystal polymer (ULTRALIM 3850)	0.0025	2.9	0.1
PDMS with glass microsphere	0.014	1.85	—
Polyimide	0.005	2.91	0.2
PDMS	0.02	2.65	—
PDMS with silicate microsphere	0.02	2.45	—
PDMS with phenolic microsphere	0.022	2.24	—
Paper (Kodak photo paper)	0.05	2.85	0.254

**Table 2:** Comparison table of various antennas with different substrates from the existing literature.

Antenna	Conductive Element	Substrate	Application	Dimensions mm^3^	Resonant Frequency Bandwidth (FBW%)		Antenna Gain and Efficiency		Ref.
					Under Normal Condition	Under Bending	Normal Condition	Under Bending	
CPW design	Silver nanoparticle-based radiating element	Polyethylene terephthalate (PET)	Wearable navigation devices	46 × 4 × 0.04	1.8 GHz and 190.5 MHz	-	2.72 dBi and 93.33%	-	[3]
Rectangular	Electro-textile material	Polyester	GPS tracking devices	40 × 30 × 1.4	1.575 GHz and 200 MHz	-	3 dBi and 80%	-	[4]
Folding antenna with double layer	Copper	Polyimide	Wireless transmission system	5 × 3 × 0.1	2.45 GHz, 5.2 GHz, 5.8 GHz	-	1.65 dBi, 4.37 dBi	-	[11]
Aperture-coupled	Copper	FR4 PCB	Wearable devices	10 × 10 × 1.4	2.45 GHz and 0.08 GHz	-	6 dBi and 47%	-	[26]
Circular patch	Copper sheet	Textile	Body-centric wireless communication	30 × 40 × 0.75	3.1–12 GHz and 7.5 GHz	3–8 GHz and 5 GHz	4 dBi and 80%	3.5 dBi and 70%	[29]
Serpentine structures	Copper	Flexible PCB Rogers RT 5880	ISM band	78 × 40 × 0.254	900 MHZ, 2.45 GHz and 220 MHz, 570 MHz	-	1.85 dBi, 2.2 dBi and 93%, 85%	-	[30]
Leaky wave antenna	Copper	Rogers RT 5880	Vital sign detection	95.4 × 46 × 2.9	60 GHz and 8 GHz	-	24.3 dBi and 95.5%	-	[46]
Coplanar waveguide fed patch antenna	Copper	FR4	Wireless application	25 × 25 × 1.4	2.45 GHz, 4.5 GHz, 5.8 GHz and 0.5 GHz, 1 GHz, 0.3 GHz	-	-	-	[49]
Frequency selective structure (FSS)	Copper sheet	Acrylic fiber sheet	Healthcare applications	15 × 15	5.45 GHz and 590 MHz	-	3 dBi and 75%	-	[50]
T-shaped antenna with electromagnetic band gap ground plane	Copper	Denim	Wristband application	35.4 × 82.4 × 40	2.45 GHz and 0.2 GHz	-	7.46 dBi	_	[58]
Circular patch	MXene film	Polydimethylsiloxane	Body motion sensor, 5G IoT	Diameter 25 mm	4.8 GHz	-	-	-	[70]
Monopole radiator with rectangular slot	Copper	Polydimethylsiloxane composite		24 × 28 × 1.52	3.125 to 13.24 GHz and 10.115	3.1 to 13.02 GHz and 9.92 GHz	2 to 4 dBi	3 dBi	[74]

## Data Availability

Not applicable.
